# Can directing self-enhancement from social to performance settings alleviate narcissistic personality disorder? Implications from narcissism-performance research

**DOI:** 10.3389/fpsyt.2026.1621098

**Published:** 2026-05-26

**Authors:** Shuge Zhang

**Affiliations:** 1 College of Physical Education, Hunan University of Technology, Zhuzhou, China; 2 Centre for Sport and Psycho-Social-Behavioural Research, Zhuzhou, China

**Keywords:** fantasy migration, narcissism, performance orientation, self-enhancement, treatment

## Introduction

The term “narcissism” originates from Ovid’s adaptation of a Greek myth where Narcissus falls in love with his reflection and dies by the waterside. It describes an obsession with self-love, aligning with Freud’s early view of narcissism as key to interpersonal regulation – that is, narcissists prioritize self-enhancement over intimate connections, manipulating relationships for personal gain (1, 2). Such a conceptualization reflects traits like self-aggrandizement, entitlement, dominance, and manipulation, which mirror contemporary theorizing of narcissism underpinned by self-centered characteristics such as self-aggrandizement, entitlement, dominance, and a manipulative orientation (3, 4).

After Freud, Kohut (1968) introduced the more extreme form of narcissism, namely *Narcissistic Personality Disorder* (NPD) (5). According to the Diagnostic and Statistical Manual of Mental Disorders (DSM-5), NPD involves a pervasive pattern of grandiosity and fantasy, a need for admiration/entitlement, a lack of empathy, and socially maladaptive interpersonal regulation (6). While research has purposefully distinguished *narcissistic personality* (i.e., a trait that exists in normal people and assessed on a continuum) from NPD (i.e., a clinical condition that requires specific diagnostic criteria to be met) (7–12), it is noteworthy that both of them share the fundamental psychological characteristics of narcissism although normal individuals with narcissism do not pervasively “require” admiration nor “pre-occupied” with grandiose fantasies to the same extent as those with NPD (13–15). As such, research on sub-clinical narcissism is relevant and can be comparable to that on NPD.

## Treatment for NPD: current approaches and limitations

Indeed, varied interventions and treatment approaches exist for the NPD. For instance, a recent systematic review (16) has identified *cognitive behavioral therapy* (e.g., addressing distorted thinking and proximal behavioral patterns associated with NPD), *psychodynamic therapy* (e.g., addressing subconscious, underlying emotions and their origins in childhood or early-year experiences and the related unresolved conflicts that impact one’s patterns of thinking and behaviors) and *schema therapy* (e.g., resolving unhealthy coping or interpersonal mechanisms developed in response to unmet emotional needs in childhood or early-year experiences) as three predominate approaches to NPD treatment. Despite these varied approaches, Weinberg and Ronningstam’s narrative review (17) has highlighted five principles underpinning effective therapies for the NPD, including ‘set realist goals’, ‘attend to treatment frame’, ‘attend to relationships and self-esteem’, ‘build alliance’, and ‘monitor countertransference’.

Reservations are, however, due to the self-esteem dysregulation central to the NPD (18), individuals with NPD are naturally resistant to any treatment (e.g., high dropout) and lack the motivation to change. This is also because attending to treatment and acknowledging their problems challenge NPD’s overly inflated self-view, and the lack of empathy explains why individuals with NPD rarely see their patterns of thinking and behaviors as problematic (19). These manifestations of the NPD lead to high dropout rates in psychotherapy and other relevant interventions or treatments (20). Moreover, building a therapeutic alliance is key to the effectiveness of NPD treatment (17). But the pervasive tendency towards manipulation and the lack of genuine empathy in individuals with NPD can result in a less effective therapeutic process. NPD’s characteristics challenge the quality of therapeutic alliance, especially when a therapist feels undermined or devalued and becomes pessimistic about a vision toward positive changes in NPD-related symptoms (21). In support of this view, research has demonstrated that individuals with NPD tend to provoke powerful feelings in their therapists, contributing to stalemates (17). Meanwhile, psychotherapists’ personality traits are associated with therapeutic orientation and interpersonal skills, which subsequently influence the therapeutic alliance and treatment outcomes (22). Thus, building a strong and lasting therapeutic alliance is not necessarily achievable or realistic. Additionally, individuals with NPD drop out of therapy or see the re-emergence of symptoms after therapy (17, 21). This is likely due to the existing approaches to treatment for NPD that have focused on reducing NPD symptoms (e.g., cognitive and interpersonal style) rather than its underlying psychological needs for self-enhancement contributing to the disorder. Addressing the pervasive and desperate needs for self-enhancement, therefore, is fundamental to alleviating NPD. However, current approaches to intervention and treatment are limited to tackling NPD’s underlying psychological needs for self-enhancement.

## Narcissists in performance settings: the ‘bright’ side of self-enhancement

Interestingly, while a pervasive tendency of seeking out opportunities for self-enhancement (e.g., admiration, entitlement, dominance) via social domains is what psychotherapy for NPD aims to alleviate and can explain why NPD (and narcissism in general) is seen problematic (23), these socially maladaptive features appear to be more adaptive than their first impression in performance settings (24). Performing to a high standard is crucial in competitive sport and other high-performing settings (e.g., performing arts, military), and thus these performance arenas naturally offer exclusive opportunities for glory or being exceptional (25). While the desire to win and the high-level competitiveness can evoke negative emotions for some, such challenges appear more appealing than threatening to individuals seeking opportunities for self-enhancement (24). In other words, the performance setting is particularly attractive to narcissists because it allows them to realize the strong desire to lionize the self and receive admiration.

Importantly, research has supported the position that the socially considered maladaptive or dysfunctional aspects of narcissism and/or NPD, such as self-assertiveness and dominance orientation (20), tend to play adaptive roles in performance settings. In their seminal work, Wallace and Baumeister (2002) proposed that narcissists, driven by a strong desire for self-enhancement, are more likely to excel in scenarios that offer opportunities for glory or social comparison but struggle in situations where such opportunities are absent (7). Through four experimental studies, they compellingly showed that narcissists outperformed their peers in high-stakes situations that allowed for self-enhancement (e.g., providing rewards, leaderboard, challenging tasks). Conversely, their performance declined significantly in low-stakes contexts lacking opportunities for self-enhancement. This work, together with its replications in varied performance domains (10, 26–28), suggests that narcissists are more capable of performing under tough and challenging situations than their counterparts, thanks to their pervasive drive for self-enhancement and personal acclaim.

Evidence also exists revealing that narcissists, especially those high in both self-assertiveness (the so-called ‘adaptive’ narcissism) and dominance orientation (the so-called ‘maladaptive’ narcissism) aspects of narcissism (29, 30), do not tolerate greater psychological challenges as a cost for performance enhancement. Specifically, Zhang et al. (2020) found that narcissistic golfers (especially those high in maladaptive narcissism) reduced their pre-putt time (i.e., the time spent in preparation for executing the putt) when performing under high-pressure, high-threat conditions (i.e., rewards, leaderboard, and feedback inducing performance-related ego threats), but their counterparts increased pre-putt time under the same conditions (31). The findings suggest that narcissistic golfers suffer less and regulate task processes better under performance pressure. In another laboratory test with college students adopting manipulations similar to the putting task, Zhang et al. (2020) further assessed narcissistic individuals’ beat-to-beat heart rate variability, measured by r-MSSD or the root mean square of successive normal-to-normal intervals as a psychophysiological marker of cardiac vagal control reflecting affective regulation and attentional control during task performance (32–34). The findings revealed that an increase in narcissistic characteristics was associated with a greater reduction in r-MSSD (reflecting superior affect and attentional regulation) under high-pressure, high-threat conditions, especially when the maladaptive, dominance aspect of narcissism was at high levels. Zhang et al. (2020) concluded that, despite often leading to interpersonal issues in social contexts (30, 35), narcissists’ willingness to dominate and the underlying desire for self-enhancement are considered ‘healthier’ in performance contexts, because such narcissistic characteristics bring performance advantages to narcissists and make them more appealing (in face of the performance demand).

More recent research has offered further insights into the adaptive role of narcissism in leadership (coaching), given an overarching performance context. Compared to leadership research from social/interpersonal perspectives that supports the emergence but not the effectiveness of narcissistic leaders (12, 36, 37), performance-focused research has shown that narcissistic leaders (coaches) appear to benefit their followers (38, 39). In three social experiments, Nevicka et al. (2013) demonstrated that followers tended to choose high narcissists as leaders in situations of uncertainty (i.e., instability and unpredictability of company outlook), even though they were aware of narcissistic leaders’ dominance orientation and the associated arrogance and exploitativeness (38). Narcissistic leaders’ undesired personal characteristics (from social/interpersonal perspectives) appeared beneficial to their followers, attenuating their sense of insecurity under uncertain times that evoke a high demand for exceptional performance (38). In another study of national-level rowers from 52 competitive rowing clubs, Nevicka et al. (2023) assessed rowers’ perceptions of their coaches’ narcissism, their perceived self-enhancement opportunities, and performance across a series of competitions at the Dutch National Championship (39). The findings revealed that increased athletes’ perceptions of coach narcissism predicted better championship performance, and this relationship was underpinned (explained) by increased athletes’ perceptions of self-enhancement. That is, athletes perceive greater opportunities for self-enhancement and perform better as their coaches’ narcissism increases (39).

While research supported that the performance settings dovetail well with narcissists’ self-enhancement (e.g., providing personal glories or self-enhancing opportunities that are rare in social or interpersonal contexts), it is noteworthy that the aforementioned research evidence is from a sub-clinical population using NPI-based narcissism assessments (i.e., Narcissistic Personality Inventory) (40–42), rather than clinically diagnosed individuals with NPD. Unlike sub-clinical narcissists, individuals with NPD are more obsessive with ‘requiring’ a sense of entitlement and admiration and preoccupied with intense fantasies that construct and maintain an inflated self-image (14, 15). They often demonstrate an extreme form of dispositional characteristics in subclinical narcissists, reflecting greater functional impairment and vulnerability (e.g., socially maladaptive behaviors, hypervigilance to threats) (13, 43). Interestingly, the vulnerable aspects of NPD have also received considerable attention in subclinical, performance settings, utilizing the subclinical assessment of vulnerable narcissism, such as the Hypersensitive Narcissism Scale (HSNS) (44) and the Five-Factor Narcissism Inventory (FFNI) (45). What more important is that studies in sport and performance settings adopting the HSNS and/or FFNI also supported the view that performance settings are more adaptive than maladaptive to individuals high in narcissism, regardless of their vulnerability.

For example, in three experimental studies of over 900 participants with varying levels of narcissism from diverse backgrounds (11), Manley et al. (2019) found that a positive association between narcissistic self-enhancement and goal-driven persistence, of which such an effect was only evident when the vulnerable aspects of narcissism were present. In other words, the vulnerable aspects of narcissism appeared to serve as a catalyst to reinforce the adaptiveness of narcissistic self-enhancement when clear performance goals can be identified (24). Compared to frequently reported and agreed pitfalls of narcissistic vulnerability in social and interpersonal domains (e.g., aggressive, counterproductive behaviors) (46–49), in a cross-sectional sample of over 200 (semi-)professional football players and a three-way longitudinal sample of over 300 competitive athletes from mixed sports (25), evidence did not support the association between narcissistic vulnerability and anti-social behaviors in sport, nor the predictability of narcissistic vulnerability in athletes’ trajectories of anti-social behaviors over a 8-month period that roughly captured a sporting season, after controlling for demographics and narcissistic grandiosity. The findings revealed that the vulnerable aspects of narcissism, often rooted in individuals with NPD, may not be as problematic in sport and performance as they are in social and interpersonal settings. Altogether, the existing evidence from subclinical narcissism research in sport and performance suggests that narcissists appear to bring more advantages than problems in performance compared to social and interpersonal settings.

## Directing self-enhancement from social to performance settings: a promising approach?

Based on the narcissism-performance research to date, this article calls for attention to the position that narcissists can glean benefits from the performance settings and transfer to mitigate their maladjustments in social contexts. Research has demonstrated that narcissists tend to behave more aggressively or in a more problematic way when facing ego threats in social contexts, due to their overwhelmed fantasy and inflated self-image being threatened and unlikely to be realized in social and interpersonal settings (50). However, the performance setting differs in that it naturally provides opportunities to be exceptional (e.g., beat an opponent, perform when others cannot, receive admiration or attention) (24). It offers narcissists ‘permitted’ or ‘socially desired’ means for self-enhancement (e.g., beating a competitor or opponent), which are structured and constrained by clear rules and controllable/adjustable performance goals, in contrast to social and interpersonal contexts. By satisfying their core psychological needs in performance settings, individuals high in narcissism (or with NPD) are likely less reinforced to seek self-enhancement via social means (e.g., dominance, self-arrogance, exploitativeness), because performance settings provide greater opportunities for a positive reinforcement that they can foresee glory that is deemed safe and certain. Such a strategy may be considered ‘*performance-oriented fantasy migration*’ as it emphasizes shifting self-enhancement and the relevant fantasies from social to performance domains. Given ego threat may be better dealt with and the sense of glory may be more fulfilling in performance compared to social settings (8), one would consider performance-oriented fantasy migration allowing narcissists to seek out self-enhancement via performance means, and thus, these narcissists may be less likely to seek self-enhancement via undesired social means, because performance settings offer them a greater opportunity for glory and maintaining the self-esteem.

Despite a sound conceptualization, one could argue that such a ‘fantasy migration’ may provide a new stage for NPD-related symptoms and disorder, rather than treating or alleviating them. That is, if self-worth or self-enhancement in individuals with NPD becomes entirely dependent on beating an opponent, winning a competition, or being an exceptional performer, would this reinforce the fragility of self and amplify pathological aspects of NPD? Such a concern is not without a reason, and no conclusive remarks can be made until the ‘*performance-oriented fantasy migration*’ is comprehensively examined empirically. Nevertheless, from what we know from the existing subclinical narcissism-performance research, the concern that ‘*performance-oriented fantasy migration*’ may reinforce the maladaptiveness of NPD is relatively low. In two samples of competitive athletes (over 700 participants in total) (51), Boulter et al. (2022) examined narcissism, intragroup conflict, and task cohesion within sport teams that varied in sport type and level of competitiveness. Although the results supported a negative relationship between athletes’ narcissistic traits and task cohesion within the sport teams, both studies consistently found that, this negative effect or undesired outcome associated with narcissism subsequently weakened as the narcissism group composition increased (i.e., assessed by an aggregation on individuals’ roundtable perceptions of the number of teammates who are considered narcissists or high in narcissistic traits). More interestingly, Boulter et al. (2022) demonstrated that, in the sporting contexts, narcissism was related to impaired task cohesion and the underpinning increased process conflict within a team (i.e., disagreement of ways of doing things as a team) alone, not creating more relationship conflict (i.e., interpersonal friction amongst a team) nor task conflict (i.e., arguments about task choices and the related outcomes for the team). These findings suggest that an increase in narcissism and a greater narcissism composition within a team, which are considered an increased risk in social settings, do not necessarily cause more problems to a sport team. Similar evidence exists, supporting that narcissism in sport team settings is linked to greater effort and commitment to the training and performance compared to their counterparts, especially when paired with coaches high in transformational leadership (51), exposed to a performance-oriented motivational climate (52), and having teammates who are also high in narcissism (53). Overall, it is highly convincing that the risk of reinforcing interpersonal functional impairments and socially maladaptive behaviors through participation in sport and performance is low.

## Theoretical underpinnings and boundary conditions

To provide further theoretical underpinnings for the premise that fulfilling self-enhancement in performance domains likely reduces (rather than increases) maladaptive self-enhancement in social domains, one can draw on Self-Affirmation Theory (54) and the principles of fluid compensation (55). According to the Self-Affirmation Theory, individuals are motivated to protect their global sense of self-integrity (i.e., an individual’s overall sense of being). When one’s self-integrity is threatened, an individual often resorts to defensive behaviors for compensation; however, if the individual can affirm self-worth in an alternative, valued domain, the need or tendency for defensiveness in the threatened domain can be significantly attenuated (54). Similarly, in the Meaning Maintenance Model (55), the proposition of fluid compensation suggests that one can restore a sense of meaning in a different domain, alternative to that when the individual experiences a meaning violation or threat.

In the context of NPD, indeed, the intense drive for interpersonal dominance, exploitation, and other socially maladaptive behaviors is largely a defensive mechanism deployed to regulate a deeply fragile and dysregulated self-esteem (18). By securing a reliable, structured source or supply of “narcissistic fuel” (e.g., opportunities for admiration, entitlement, and a sense of dominance) within a performance setting, the individual’s global self-worth is likely to be affirmed (7, 56). Consequently, the psychological need or a state of urgency to achieve self-enhancement via manipulative, aggressive, or other maladaptive social means is theoretically satiated, or at least largely reduced, according to the Self-Affirmation Theory (54) and the principles of fluid compensation (55).

While direct clinical evidence supporting this specific domain-shifting self-affirmation in the NPD population is pending, compelling empirical evidence supports this fluid compensation mechanism. For instance, in a seminal experimental study (57), Thomaes et al. (2009) demonstrated that when narcissists were guided through a brief self-affirmation exercise (i.e., reflecting on a core personal value), their subsequent aggressive and maladaptive responses to an ego threat were markedly diminished compared with those in the control group. Moreover, research by Giacomin and Jordan (2014) revealed that temporarily boosting or affirming a narcissist’s sense of security has been shown to reduce their characteristic sense of psychological entitlement and interpersonal defensiveness (58). Importantly, the findings suggested that the state-like manner of narcissism, such as the mutually reinforcing intra- vs. inter-personal processes (e.g., fragility vs exploitativeness (59–61);, is more context-dependent than previously assumed. In four studies, Giacomin and Jordan (2014) consistently demonstrated that narcissists’ agentic concerns and state-like manner can be altered via increasing communal focus or vice versa. If temporary contextual shifts and brief psychological interventions can successfully down-regulate maladaptive narcissistic states and interpersonal defensiveness, it is highly plausible that a sustained, strategically structured approach, such as ‘performance-oriented fantasy migration’, could yield potential therapeutic benefits. By systematically channeling narcissists’ intense agentic needs and desires for self-enhancement into a dedicated, rule-bound performance domain, the psychological urgency to exhibit dominance, entitlement, and exploitativeness in everyday social and interpersonal settings is likely to be significantly attenuated.

However, a crucial boundary condition for the ‘performance-oriented fantasy migration’ arises when an individual experiences persistent failures or significant setbacks in the performance setting. Given the inherent fragility of narcissistic self-esteem (18), one may come up with the concern that a prolonged lack of success could trigger substantial ego threats, potentially leading to disengagement from the performance domain and a reversion to socially maladaptive strategies where reality can be more easily manipulated via narcissistic means to preserve an (overly) inflated self-image. Such a concern is legitimate, suggesting that the ‘performance-oriented fantasy migration’ should not be a standalone intervention or practice but operate as a strategic adjunct to ongoing psychotherapy, with the therapeutic alliance being paramount (17). In this context, the goal of ‘performance-oriented fantasy migration’ is not merely to satisfy a narcissist’s desperate need for admiration and entitlement via winning, but to engage narcissists meaningfully with the process of achievement and growth naturally grounded in a structured performance setting, thus fulfilling self-enhancement via a healthier and more socially acceptable or desirable means (e.g., exerting effort, excelling oneself).

Indeed, empirical research, even in the subclinical population, supported that while narcissists are highly sensitive to ego threats such as performance failure feedback, their response to performance setbacks is not withdrawal; instead, they tend to be more persistent, give greater effort, and appear more self-assertive in task performance (10, 11, 31). For example, with everything else equal, in a puzzle-solving task, Manley et al. (2018) found that high narcissists tend to persist in solving the tasks or give up after experiencing more failure trials compared to their counterparts (11). Similarly, Roberts et al. (2019) demonstrated in both a fine motor control task and an enduring task that narcissists appeared to exert more effort regardless of performance when provided with high-performance pressure and rarely achievable tasks (10). Furthermore, in two laboratory-based studies of golf-putting competition and cognitive performance tasks, Zhang et al. (2020) observed reduced reaction time and pre-task routine (i.e., indicating greater task efficiency and lowered hesitation) and increased root mean square of successive normal-to-normal intervals (r-MSSD; a psychophysiological indicator of superior affect regulation) (31). Field study on narcissism in athletic training also revealed that an increase in narcissism was associated with greater coping with adversity, especially when the opportunity for (even a future) success is articulated (9). These findings suggest a capacity for persistence and adaptation in performance-oriented narcissists when they believe conditions for performance success exist.

Reimaging this integrated therapeutic context (i.e., using ‘performance-oriented fantasy migration’ as a therapeutic hook), the structured nature of the performance environment, characterized by its “agreed-upon rules and standards” (see more below in the practical considerations), becomes a vital asset. Unlike the ambiguity of social interactions, objective performance metrics minimize opportunities for narcissistic distortion of one’s actual capabilities. A skilled therapist would then better utilize both successes and, critically, failures as potent teachable moments within the secure therapeutic space. The therapeutic process, therefore, would actively guide the individual to interpret performance failures not as proof of their worthlessness but as instrumental feedback for improvement and ultimate success, thereby fostering genuine internal stability and resilience rather than reinforcing maladaptive compensation. ‘Performance-oriented fantasy migration’ presents an opportunity for therapists to cultivate a resilient, adaptive sense of self-worth that withstands external challenges and can be learned regardless of performance success or setbacks.

## Moving forward: practical considerations and recommendations for implementation

Nevertheless, ‘performance-oriented fantasy migration’ has yet to be examined empirically in clinical population with NPD. This opinion, therefore, calls for the consideration of the proposal and encourages a wide community of researchers and practitioners to test and examine this proposal as appropriate. To facilitate researchers and practitioners in their future works, this opinion proposes four practical considerations for incorporating the ‘performance-oriented fantasy migration’ principle into intervention and treatment for NPD.

First, identify or create a meaningful performance setting for fulfilling self-enhancement. The performance setting where narcissists can glean self-enhancement benefits must be relevant and meaningful to them. Without a meaning (e.g., a real context that matters), dominating or becoming exceptional is meaningless and thus in no way satisfying to a narcissist’s inflated self. Second, ensure an agreement on performance rules and standards. This is because unclear or indecisive performance rules and standards increase narcissists’ self-handicapping or taking advantage of any ‘grey’ rule (62), which in turn may reinforce their maladaptiveness in social domains. With agreed (and unshakable) performance rules and standards, narcissists may be more likely to seek out self-enhancement via performance (e.g., beating the opponents following performance rules), not social means (e.g., establishing dominance or sense of control via aggressive or exploitative ways).

Moreover, identify structured competitive or mastery domains for performance-oriented self-enhancement. While competitive sports (e.g., individual- or team-based sports or other competitive activities) offer highly structured environments where performance can be objectively measured and appreciated, providing narcissists with ‘healthy’ forms of admiration and an opportunity to foster interpersonal skills through social interaction and conflict-solving (63, 64), it is recognized that traditional sports may not be universally accessible or appealing to all individuals with NPD. Factors such as physical limitations, lack of interest, or past negative experiences could render conventional sporting contexts ineffective or inapplicable. Therefore, the principle of ‘performance-oriented fantasy migration’ extends to any performance domain that offers similar contexts (e.g., clear rules, objective achievement metrics, opportunities for skill mastery) for healthier means of self-enhancement. Such domains could include competitive video gaming where rank and skill are objectively defined (e.g., esports), strategic board games like chess, structured artistic pursuits (e.g., competitive music performance, advanced crafts with judged outcomes), or intellectually demanding hobbies (e.g., coding challenges, academic competitions). The crucial elements across all these settings are the provision of tangible, objective performance feedback and clear criteria for success, which serve as concrete and legitimate avenues for self-enhancement. Diversifying the accessible performance domains opens therapeutic opportunities for fantasy migration, tailored to a broader range of individual interests and abilities across varied performance contexts. Further research would benefit from exploring the efficacy and optimal engagement strategies across these varied domains for individuals with NPD.

Lastly, monitor potential risks and reinforce good practices via reflective dialogue. Performance-oriented fantasy migration may lead to the desired benefits (i.e., fulfill self-enhancement via healthy means) only when adaptive performance-related behaviors (e.g., confidently taking the responsibility in difficult performance) and cognitions (e.g., I am great because I dare to strive when others fear to) are fostered through proper reflection. Practitioners should commit to positive reinforcement, taking advantage of the performance settings to help narcissists shape a healthy alternative to self-enhancement, whilst monitoring the potential risk of transforming the inflated self from performance into social domains. While the ‘performance-oriented fantasy migration’ framework proposed in this opinion piece stands strong from a psycho-behavioral perspective, researchers and practitioners should consider incorporating neurobiological or psychophysiological markers as a monitoring approach to assessing therapeutic efficacy, as research has demonstrated considerable evidence of distinguishing affect and emotional regulation underpinning NPD using varied neural and physiological markers (65–67).

In light of the important principles of implementing ‘performance-oriented fantasy migration’, future research should operationalize and test the efficacy of this ‘fantasy migration’ framework through a randomized controlled pilot trial involving individuals clinically diagnosed with NPD. Participants could be randomly assigned to either a standard treatment control group (i.e., receiving treatment as usual) or a “fantasy migration” intervention group. The intervention group would receive standard psychotherapy combined with an adjunctive, structured competitive sports program alongside the therapeutic practices. In this experimental condition, the agreed-upon rules of the sport would provide the necessary boundaries, while concurrent therapy sessions would use reflective dialogue to process the patient’s performance outcomes, treating both victories and setbacks as teachable moments to build internal emotional stability rather than fragile grandiosity. Such an approach is considered low risk because it operationalizes ‘fantasy migration’ whilst simultaneously providing patients with the standard or routine therapies that are considered to some extent effective.

To comprehensively evaluate the therapeutic efficacy of this approach, the pilot study or the clinical implementation should assess a combination of clinical, behavioral, and biological outcomes. Clinically, researchers could track therapy dropout rates, the strength of the therapeutic alliance (e.g., using the Working Alliance Inventory), and changes in socially maladaptive behaviors over time. Crucially, building upon the psychophysiological monitoring mentioned above, the study could assess baseline and post-intervention heart rate variability (e.g., r-MSSD) or cortisol reactivity during standardized social stress tasks. By capturing these objective neurobiological markers alongside behavioral data, such a design would provide robust, measurable evidence of whether ‘performance-oriented fantasy migration’ successfully reduces internalizing symptoms and emotional dysregulation in NPD, thereby moving the proposed framework from conceptual observation to evidence-based clinical intervention.

## Conclusion

In sum, current approaches to NPD treatment are generally limited by resistance to therapy, poor therapeutic alliance, and a focus on symptom reduction rather than psychological underpinnings. While NPD (and the underlying narcissism) is often viewed as maladaptive in unstructured social contexts, evidence suggests that narcissistic traits are highly compatible with performance settings. By incorporating the principle of ‘performance-oriented fantasy migration’, interventions for NPD could channel the core psychological need for self-enhancement into structured, attention-evoking, and rule-bound competitive environments. Crucially, this approach does not simply relocate the pathology; rather, it utilizes the performance domain as a therapeutic hook. By processing both victories and setbacks within a secure therapeutic alliance, practitioners can help individuals with NPD build genuine internal emotional stability and mitigate the vulnerable, fragile aspects of their self-esteem. Furthermore, incorporating neurobiological markers (e.g., HRV, cortisol) alongside behavioral outcomes in future pilot studies is vital to moving this concept from behavioral observation to an evidence-based clinical intervention. This opinion, therefore, calls for researchers and practitioners to test the proposed framework and examine the multidimensional efficacy of ‘performance-oriented fantasy migration’ in NPD treatment.
